# Rural job preferences of graduate class medical students in Ethiopia—a discrete choice experiment (DCE)

**DOI:** 10.1186/s12909-023-04133-3

**Published:** 2023-03-13

**Authors:** Mamo Engidaw, Melaku Birhanu Alemu, Getasew Amare Muche, Mezgebu Yitayal

**Affiliations:** 1 Amhara Development Association, Woldia, Amhara Ethiopia; 2 Department of Health Systems and Policy, Institute of Public Health, University of Gondar, Gondar, Ethiopia

**Keywords:** Discrete choice experiment, DCE, Medical students, Job preference, Ethiopia

## Abstract

**Background:**

Human resource is one of the health system’s building blocks, which ultimately leads to improved health status, equity, and efficiency. However, human resources in the health sector are characterized by high attrition, distributional imbalance, and geographic inequalities in urban and rural settings.

**Methods:**

An discrete choice experiment (DCE) with 16 choice tasks with two blocks containing five attributes (salary, housing, drug and medical equipment, year of experience before study leave, management support, and workload) were conducted. A latent class and mixed logit model were fitted to estimate the rural job preferences and heterogeneity. Furthermore, the relative importance, willingness to accept and marginal choice probabilities were calculated. Finally, the interaction of preference with age and sex was tested.

**Results:**

A total of 352 (5632 observations) final-year medical students completed the choice tasks. On average, respondents prefer to work with a higher salary with a superior housing allowance In addition, respondents prefer a health facility with a stock of drug and medical equipment which provide education opportunities after one year of service with supportive management with a normal workload. Young medical students prefer lower service years more than older students. Besides age and service year, we do not find an interaction between age/sex and rural job preference attributes.

A three-class latent class model best fits the data. The salary was the most important attribute in classes 1 and 3. Contrary to the other classes, respondents in class 2 do not have a significant preference for salary. Respondents were willing to accept an additional 4271 ETB (104.2 USD), 1998 ETB (48.7 USD), 1896 ETB (46.2 USD), 1869 (45.6 USD), and 1175 ETB (28.7 USD) per month for the inadequate drug and medical supply, mandatory two years of service, heavy workload, unsupportive management, and basic housing, respectively.

**Conclusion:**

Rural job uptake by medical students was influenced by all the attributes, and there was individual and group-level heterogeneity in preference. Policymakers should account for the job preferences and heterogeneity to incentivize medical graduates to work in rural settings and minimize attrition.

**Supplementary Information:**

The online version contains supplementary material available at 10.1186/s12909-023-04133-3.

## Background

Human resource is one of the building blocks of a health system, which ultimately leads to improved health status, equity, and efficiency [1]. A variety of national and international policy and strategy documents as a vital input for the health sector in resource-limited countries have extensively recognized it. In developing countries, the efforts to solve human resource problems have not achieved the desired results [2].

There is a shortage of physicians, nurses, and other health workers to deliver essential health services worldwide [3, 4]. The World Health Organization (WHO) estimated a projected deficit of 18 million health workers by 2030. Most of the shortfall was predicted in low and lower-middle-income countries [5]. In addition, there are distributional inequities in the availability of healthcare workers across countries and within countries [6, 7].

There is a maldistribution of the workforce towards urban jobs. America has 37% of the total health workforce but has only a 10% disease burden, whereas Africa has only 3% of the health workers working in Africa with a 25% global disease burden [3, 6–11]. Africa, particularly sub-Saharan Africa, with a rapidly growing population, has the lowest health workforce-to-population ratio. With 85% of the population living in rural areas, Ethiopia has skewed health workforce distribution in favour of urban settings n resides [12–16]. Despite the desperate demand for medical doctors, especially in rural and remote, under-served areas, many professionals from developing countries are moved to developed countries [17]. Ethiopia lost more than two billion dollars from training physicians that migrated to Australia, Canada, the United Kingdom, or the United States [17, 18].

Different studies identified several factors affecting graduate medical students' and other health professionals' job choices/preferences. Allowance/salary, refreshment training, human resource management style, facility quality [19–22], age, marital status, years of experience, sex, place of birth, previous rural exposure, and family income [23–27] were among the factors that affect the physicians job preference.

Ethiopia is among the countries with very low physicians among the sab-Saharan African countries. The country designs a flooding or rapid scale-up strategy to produce medical doctors in different public health universities and hospitals. The medical education curriculum is nationally harmonized and the training is provided in the approaches or platforms. The conventional/genetic one trains a 12-grade completed and newly enrolled students for six years. The second approach is the innovative one, where first-degree graduates with science disciplines are recruited after two years of minimum experience and trained for four years. After completing the training course and graduation, the medical doctors are assigned to the area needed by the health ministry through a lottery method [28].

The Ethiopian Federal Ministry of Health (FMoH) set the Health Sector Transformation Plan (HSTP) to improve equity, quality, coverage, and utilization of essential health services in all segments of the country that depends on the convenience of the acceptable number and skilled professionals [29]. Achieving HSTP is a challenge because of the largely skewed distribution of physicians and specialists towards the urban areas and the dis-proportionality of nursing and medical school graduates entering the urban labour market after graduation [30]. In 2010, only 7% of physicians were willing to take a rural job over a job in the capital city. In addition, more than 90% of medical students and two-thirds of nursing students want to work in urban setting posts [31]. This figure, however, could change by incentivizing healthcare providers [32].

A combination of different incentives need to be designed to recruit and retain health professionals in rural and remote areas. This could be done by considering the factors influencing the health worker’s decision to work in rural or remote areas and matching the interventions with the health worker’s preferences and expectations [11]. To improve the health workforce distribution in rural and remote under-served parts of the country and to implement the transformation plan, there needs to be task shifting [33–35], educational strategies [11], strengthening financial and non-financial incentives [36], and continuous professional development [11]. However, as to the researcher’s knowledge, very little is known about the rural job preferences and preference heterogeneity of medical students. For example, the relative importance of attributes and the marginal uptake rate are unknown. Therefore, this preference study is crucial to understand final-year medical students' rural job preferences to meet their expectations. Furthermore, this research provides evidence of the probability of rural job uptake for a policy change.

A discrete choice experiment (DCE) is a novel, economical method to measure the stated preferences of an individual or a population and can be used to estimate population preference and relative importance of attributes, trade-offs between attributes, acceptability rate, and choice predictions [37, 38]. The choice experiment is founded on Lancaster’s value theory which states that utility gets from the characteristics of the object (attribute) [39], and random utility theory, which states that utility has systematic and random components [40]. Evidence showed that a DCE could accurately predict healthcare choices if it accounts for scale and preference heterogeneity [41]. It can be used to measure the health workers' job preference for certain rural practice features and quantitatively predict the job uptake, given a set of job characteristics [11, 42].

## Methods

### Study setting and period

The study was conducted in public universities in Northwest Ethiopia that give physicians generic medical training. The University of Gondar, Bahir Dar University and Debre Tabor universities have graduate students in medicine. The study was conducted from March to April 2021.

The University of Gondar (UoG), formerly known as the Gondar College of Medical Sciences until 2003, is the oldest medical school in Ethiopia. It was established as a Public Health College in 1954 in the ancient city of Gondar. The University provides different bachelor's, master's, and doctorate programs. The Bahir Dar University (BDU) was founded in the capital city of Amhara National Regional State, Bahir Dar, in 1963 as a polytechnic institute. The University comprises two schools, seven faculties, five colleges, four institutes, and two academies. Finally, the Debre Tabor University (DBTU) is a relatively new university in the Northwest region of Amhara National Regional State, established in 2008.

### Discrete choice experiment

The DCE is a quantitative technique used to assess health professionals' stated preferences (where individuals state their preferences to hypothetical choices) in the absence of revealed preference data. It is a tool for informing decision-makers on how to design strategies to address human resources problems. It is used to examine the effect of factors that influence the recruitment and retention of health workers in remote and rural regions [42, 43]. The result could be used to incentivize and attract health workers to rural areas where they needed the most. The DCE can provide information on important attributes (statistically significant), the direction of importance (the sign of the estimated parameters) and relative importance (size of the parameters), trade-off, and probability of take-up as non-monetary incentives are significant determinants of job choice [42, 44].

### Identification of attributes and assignment of levels

Six main attributes were identified from other studies through a literature review. The attributes are reviewed and evaluated by experts in the field of human resources for health, health policy, and health economics. These attributes include salary [32, 45–47]; housing [32, 45–49]; drug, equipment, and supplies [32, 45, 47–49]; years of work experience before study leave [32, 45–47, 49]; management support [47, 50] and workload [45]. The attribute levels were selected to reveal the range of working conditions students might anticipate (Table 1).

**Table 1:** Attributes and levels used for the final discrete choice experiment

Attributes	levels	Conceptual definition
**Salary**	Base salary	The base salary was 9056 ETB (220.9 USD) per month
	Base + 25% rural allowance	The base salary (9056 ETB) + rural allowance 25% (2264 ETB) = 11,320 ETB (276.1 USD) per month
	Base + 50% rural allowance	The base salary (9056 ETB) + rural allowance 50% (2264 ETB) = 13,584 ETB (331.3 USD) per month
	Base + 75% rural allowance	The base salary (9056 ETB) + rural allowance 75% (2264 ETB) = 15,848 ETB (386.5 USD) per month
**Housing**	Basic housing free of charge	two bedrooms, one bathroom, and a kitchen
	Superior housing free of charge	More than three bedrooms, two bathrooms, internet and TV
**Drug, equipment, and supplies**	Inadequate	Lower than the government standard package
	Adequate	The government standard package
**Mandatory years of experience**	Two-year rural experience	Mandatory two years of experience needed before study leave
	One-year rural experience	Mandatory one year of experience needed before study leave
**Management support**	Supportive	The management team is supportive of the clinical staffs
	Unsupportive	The management team is not supportive of the clinical staffs
**Workload**	Normal Duties	Nearly enough time to complete
	Heavy	Barely enough time to complete duties

*ETB* Ethiopian Birr; *USD* United States Dollar

### Experimental design

After identifying attributes and their levels, an experimental design was conducted to develop hypothetical DCE job scenarios. A fractional factorial design with orthogonal main effects was created by using STATA 16. Forty-eight scenarios (choice cards) were generated, and the questionnaire was written assuming two hypothetical job choices to compare them. The questionnaire consisted of twenty-four comparisons between two hypothetical jobs. Choice sets in the catalogue were randomized and categorized into three blocks (8 choice tasks in each block). Each respondent was asked to complete two blocks of choice tasks randomly.

The experimental design considers desirable design criteria: Orthogonality (attribute levels appear in choice sets with equal frequency with each level of each other attribute), level balance (levels of each attribute appear equally often), minimum overlap (minimize the overlap of levels for each attribute in each choice) and utility balance (options in each choice set have similar probabilities of being chosen) [51, 52]. Table 2 presents a sample choice scenario constructed for this research.

**Table 2:**
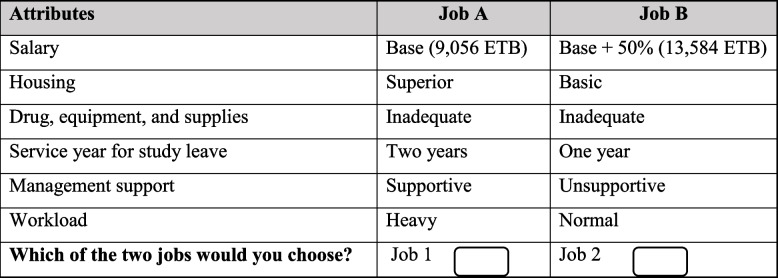
Sample job pair scenarios

### Sample size determination and sampling procedures

#### Sample size determination

There is no standard method to determine the sample size in a discrete choice experiment. However, various methods were proposed by different scholars [53–57]. A total of 352 respondents participated in this study from the University of Gondar and Bahir Dar University which makes a total of 5632 observations (352 respondents* 16 choice tasks). The sample size was sufficient for preference estimation and heterogeneity analysis based on the Johnson and Orme [55], Lancsar and Louviere [56], and Pearmain’s rule of thumb [57] recommendations.

#### Sampling procedures

There were 333, 164, and 45 graduate medical students at the University of Gondar, Bahir Dar University, and Debre Tabor University, respectively. The pilot study was conducted at the Debre Tabor University, and the final study was conducted at the University of Gondar and Bahir Dar University.

**Fig. 1:**
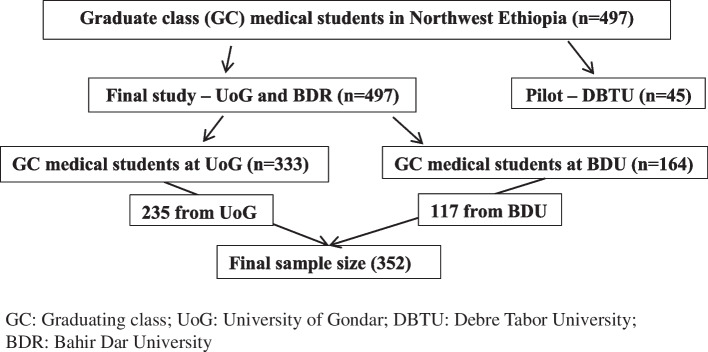
Schematic presentation of the sampling procedure

A simple random sampling technique was used to select the participants using their identification numbers. The sample was proportionally allocated to the University of Gondar and the Bahir Dar University (Fig. 1).

### Data collection and management

Structured questionnaires containing socio-demographic information, career and professional-related variables and the choice tasks were used. The questionnaire was adapted from a similar study conducted in Ghana [47] and was modified to the Ethiopian context.

The data were entered into Epi Data 3.1 software. Data cleaning was performed using frequencies and cross-tabulations to check accuracy, consistency, and missing values. The cleaned data was transferred to STATA version 16 for analysis. The data was presented using tables, figures, and charts. The frequency, mean and standard deviation of the findings were presented descriptively. Choice modelling was used to estimate the preference and heterogeneity in rural job preference. Salary was converted to the United States dollar based on the average conversion rate (1 USD = 41 ETB) in March and April 2021. I.e., Only the Ethiopian birr was presented during the choice experiment (Table 2).

### Choice modelling

A latent class model was fitted, which assesses unobserved preference heterogeneity. This method helps evaluate medical students' willingness to trade attributes in different latent classes [58]. The model assumes attributes have a heterogeneous effect and identifies unobserved classes in the response. In each class, the preference weight was estimated using conditional logit regression. The best-fitting model (appropriate number of classes) was determined using Akaike information criteria (AIC) and log-likelihood [38, 59]. The preference and relative importance of attributes was estimated in each latent class.

The relative importance of attributes was determined by subtracting the highest attribute level values from the lowest estimate in each latent class. The attribute with the largest differences value resembles the most important attribute and received a total score of 1. Their difference value was divided by the difference value of the most important attribute for all other attributes. The latent class share adjusted pooled relative importance of attributes was calculated by considering the class share.

A main effect mixed logit model (MIXL) was used to estimate the respondents' willingness to accept (salary) using 2000 Halton draws. The models allow attribute coefficients to vary across respondents, accounting for preference heterogeneity, improving the realism of model assumptions, and adjusting the standard errors of utility estimates to account for repeated choices by the same individual [60].

The utility was estimated using the following model:

$$\mathrm U(\mathrm{yes})={\mathrm\beta}_{\mathrm{salary}}\;\times\;\mathrm{Salary}\;+\;{\mathrm\beta}_{\mathrm{house}\_\mathrm{uperior}\;}\times\;\mathrm{House}\;\mathrm{Superior}\;+\;{\mathrm\beta}_{\mathrm{drug}\_\mathrm{dequate}}\;\times\;\mathrm{Dru}g\;\mathrm{Adequate}\;+\;{\mathrm\beta}_{\mathrm{serve}\_\mathrm{neyear}\;}\times\;\mathrm{Serve}\;\mathrm{one}\;\mathrm{year}\;+\;{\mathrm\beta}_{\mathrm{managment}\_\mathrm{support}}\;\times\;\mathrm{Management}\;\mathrm{Support}+{\mathrm\beta}_{\mathrm{workload}\_\mathrm{normal}}\;\times\;\mathrm{Workload}\;\mathrm{Normal}$$

The willingness to accept (salary) estimates was calculated by dividing attribute coefficients by the salary coefficient. Therefore, the attributes were compared on a common value scale in terms of willingness to accept. The confidence interval for the willingness to accept was estimated to account for uncertainty [56, 61].

The policy impact of attribute change was predicted by calculating the uptake rate change of rural jobs. The probability that respondents chose each alternative in a choice set allowed a comparison of the impact of each attribute in a common metric. This was done by keeping other attributes constant while changing one attribute level from the base case [44, 56].

## Results

### Socio-demographic characteristics of the study participants

Three hundred fifty-two medical students completed the 16 choice tasks, giving 5632 pairwise observations. Of the total respondents, 264 (75%) were males, and the mean age and standard deviation of respondents were 24 ± 1.3 years. The majority, 244 (69.32%) of respondents' parents, were from the urban part of the country (Table 3).

**Table 3:** Socio-demographic characteristics of participants in Northwest Ethiopia, 2021

Variables	Category	Number	Per cent (%)
**Sex**	Male	264	75.00
	Female	88	25.00
**Religion**	Orthodox	301	85.5
	Muslim	19	5.40
	Catholic	3	0.85
	Protestant	25	7.10
	Others	4	1.14
**Home Institution**	University of Gondar	235	66.76
	Bahir Dar University	117	33.24
**Family Residence**	Urban	244	69.32
	Rural	108	30.68
**Mother's educational level**	College diploma and above	126	35.8
	Secondary school	61	17.33
	Elementary school	75	21.31
	Unable to read and write	90	25.57
**Mother's occupation**	Housewife	199	56.53
	Self-employed	63	17.90
	Government employee	81	23.01
	Others	9	2.56
**Father's educational level**	College diploma and above	175	49.72
	Secondary school	49	13.92
	Elementary school	68	19.32
	Unable to read and write	60	17.05
**Father’s occupation**	Farming	111	31.53
	Self-employed	107	30.40
	Government employee	122	34.66
	Others	12	3.41

### Career and professional information

For the motivation of students to learn medicine, the desire to help others 173 (49.15%) and job security 117 (33.24%) were the major factors. Two hundred forty-nine (70.74%) of the students were willing to do it in rural areas, from which 154 (61.35%) the reason is to serve humanity. One in three students had outreach service experiences in rural parts of the country. More than half 184 (52.27%) of graduating medical students wanted to emigrate abroad. Majority of respondents wanted to specialize in surgery (29.55%) and internal medicine (26.99%) (Table 4).

**Table 4:** Career and professional information of medical students in selected universities, Northwest Ethiopia, 2021

Variables	Category	Number	Per cent (%)
**The original motivation to learn medicine**	Desire to help others	173	49.15
	Work internationally	26	7.39
	Research opportunities	9	2.56
	Job security	117	33.24
	Others	27	7.67
**Current motivation for learning medicine**	Desire to help others	178	50.57
	Work internationally	46	13.07
	Research opportunities	12	3.41
	Job security	84	23.86
	Others	32	9.09
**Participation in outreached service**	Yes	119	33.81
	No	233	66.19
**Reasons not to work in a deprived area**	Insufficient financial incentives	30	29.13
	Lack of training opportunities	23	22.33
	Insufficient research opportunities	3	2.91
	Poor quality of facilities	25	24.27
	Poor quality of housing	6	5.83
	Insufficient professional support	7	6.80
	Difficult to return to school	4	3.88
	Others	5	4.85
**Intention to migrate to other countries**	Yes	184	52.27
	No	168	47.73
**Expected future specialty**	Obstetrics and Gynecology	52	14.77
	Surgery	104	29.55
	Pediatrics	31	8.81
	Internal Medicine	95	26.99
	Others^*^	70	19.89

^*^Radiology, dermatology, anesthesia, psychiatry, public health, management, ENT

### Preference heterogeneity—LCM

A three-class latent class model fitted to identify unobserved preference heterogeneity. The class shares for the three classes were 29.8%, 41.7%, and 28.5%, respectively.

Respondents from class one and class three prefer rural jobs when salary increases, but respondents in class two their rural job preference insignificant for salary. Besides, class one respondents prefer better housing conditions over basic housing conditions for rural jobs. But, respondents' preference from classes two and three was insignificant for rural job housing conditions.

A one-year rural experience before the study leaves significantly affects participants' preference from class one for rural jobs over two years of rural experience. But, for respondents from classes two and three, their preference for years of work experience before study leave is insignificant for the rural job.

Likewise, class one and two respondents prefer an adequate supply of drugs and basic medical equipment over its inadequate availability for rural jobs. However, respondents from class three show that their preference for drug and basic medical equipment availability is insignificant for rural jobs.

Respondents from classes two and three prefer supportive organizational management over unsupportive ones for rural jobs. But this attribute is insignificant for class one participant's preference for rural jobs. Similarly, average workload significantly affects participants' preference from lass two and three for rural jobs over heavy workload. But, the preference of participants from class one is not affected the workload for rural jobs (Table 5).

**Table 5:** Latent class model for a rural job preference

Attribute	Levels	Class 1		Class 2		Class 3	
		**Coef**	**Std.Err**	**Coef**	**Std.Err**	**Coef**	**Std.Err**
**Salary (1 ETB = 0.0244 USD)**		0.0003078^***^	0.0000855	4.53e-06	0.0000429	0.0006824^***^	0.0001103
**Housing**	House Basic	Ref					
	House superior	0.86^***^	0.28	0.16	0.17	-0.26	0.30
**Drug, equipment, and supplies**	Inadequate						
	Adequate	1.47^***^	0.38	0.91^***^	0.17	-0.09	0.30
**Mandatory experience before a study leave**	Two years						
	One year	1.69^**^*	0.30	-0.08	0.19	-0.01	0.29
**Supportive management**	Unsupportive	Ref					
	Supportive	-0.22	0.40	0.71^***^	0.14	0.83^***^	0.26
**Average workload**	Heavy	Ref					
	Normal	0.31	0.34	0.48^***^	0.14	0.56^*^	0.29
**Class share**		29.8%		41.7%		28.5%	

*Salary was linearly coded*

^***^P-value < *0.01*

^**^P-value < *0.05*

^*^P-value < *0.10 Coef., Coefficient; Std. Err, Standard error; Ref, Reference category*

### The relative importance of attributes in rural jobs preference

**Fig. 2:**
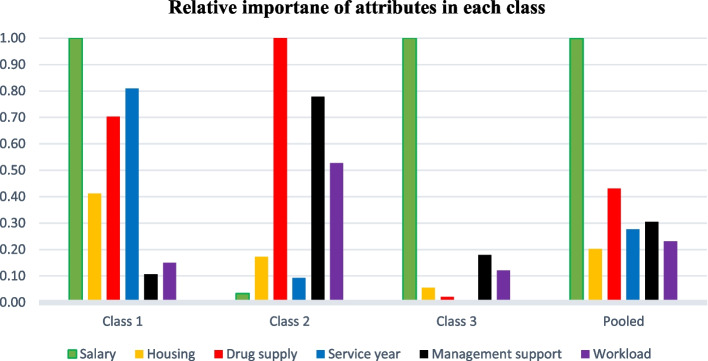
Relative importance of attributes in each class

This study observed preference heterogeneity among study participants for rural jobs. The salary was the most valued attribute for classes one and three. Relative to class 1, payment was the dominant attribute for rural job uptake for class 3 respondents. The availability of drugs and medical supplies was the most important attribute for respondents in class 2. However, it was the least important attribute for respondents in class 3. The mandatory service year before a study leave and management support were respondents' second most important attributes in classes 1 and 2, respectively (Fig. 2).

### Willingness to accept and choice probabilities – MIXL

The MIXL model showed that respondents have a significant preference variation for attributes. There was a significant preference heterogeneity for housing, drug supply, service year, and management support. However, there is no individual-level preference variation for the workload. The willingness to pay and choice probability analysis were based on the mixed logit model (Tables 6).

**Table 6:** Mixed logit model analysis of respondents

Attribute	Levels	Coef	Std.Err	SD	Std.Err (SD)
**Salary (1 Birr = 0.0244 USD)**		0.0002371^***^	0.0000248		
**Housing**	House Basic	Ref			
	House superior	0.28^**^	0.11	0.65^***^	0.12
**Drug, equipment, and supplies**	Inadequate	Ref			
	Adequate	1.01^***^	0.12	1.13^***^	0.14
**Mandatory experience before a study leave**	Two years	Ref			
	One year	0.47^***^	0.10	0.98^***^	0.11
**Supportive management**	Unsupportive	Ref			
	Supportive	0.44^***^	0.10	0.74^***^	0.14
**Average workload**	Heavy	Ref			
	Normal	0.45^***^	0.09	0.01	0.23

*Salary was linearly coded*

^***^P-value < *0.01*

^**^P-value < *0.05*

^*^P-value < 0.10 Coef., Coefficient; Std. Err, Standard error; Std.Err*SD, standard error of the standard deviation; Ref, Reference category

### Willingness to accept

**Fig. 3:**
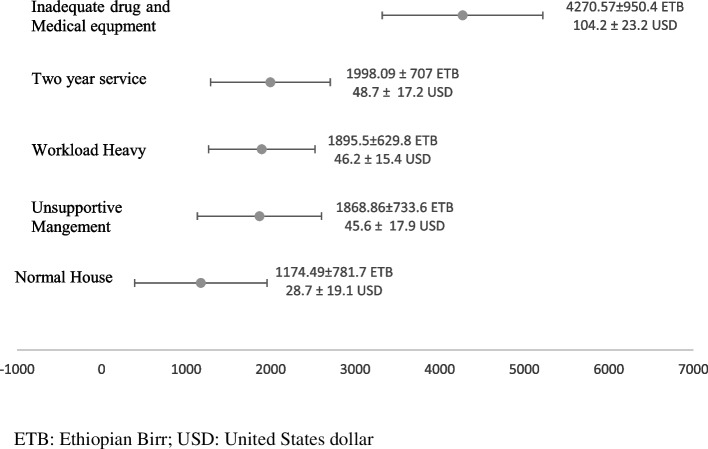
Willingness to accept the attribute levels (per month)

In this study, participants were willing to forgo 4270 ETB (104.1 USD) per month when drugs and medical supplies were adequately available. In addition, they were willing to pay 1998 ETB (48.7 USD) per month when the year of service before study leave was reduced from two to one year. Similarly, participants were willing to pay 1895 ETB (46.2 ETB) per month when the workload was normal and balanced with their time. They were also willing to pay 1688 ETB (41.2 USD) per month when the work environment and the management were supportive. For better housing conditions, participants were also willing to pay 1174 ETB (28.6 USD) per month (Fig. 3).

### Choice probabilities

A salary change has significant effect on the uptake of rural job my medical doctors. Keeping other factors (attributes) constant, a salary increase from 9056 ETB (220.9 USD) to 11,320 ETB (276.1 USD) per month would increase medical students' rural job uptake by 26.21 percentage points. Likewise, keeping other things constant, a salary increase from 9056 ETB (220.9 USD) to 13,584 ETB (331.3 USD) and 15,848 ETB (386.5 USD) per month would increase the uptake of the rural job by 48 and 66.7 percentage points, respectively. In addition, by improving the housing condition from basic to better (superior), the preference for rural jobs would increase by 13.8 percentage points, keeping other variables constant. Reducing the mandatory year of experience before a study leave from two to one year increases rural job uptake by 23.25 percentage points, ceteris paribus. Also, making the work environment safe and the management supportive would increase the rural job uptake by 21.8 percentage points, ceteris paribus. The nature of the workload would also affect the rural job uptake, where making the workload normal would increase the rural job preference by 22 percentage points, keeping other variables constant (Table 7).

**Table 7:** The marginal change of uptake rate for a change in attribute level

Base attribute	Policy changes	Uptake rate change (Δ%)	95% CI (%)	
			**Lower**	**Upper**
Salary 9056 ETB (220.9 USD)	Salary 11,320 ETB (276.1 USD)	26.21^***^	21.08	30.08
Salary 9056 ETB (220.9 USD)	Salary 13,584 ETB (331.3 USD)	49.05^***^	40.69	57.41
Salary 9056 ETB (220.9 USD)	Salary 15,848 ETB (386.5 USD)	66.69^***^	57.51	75.86
Basic house	Superior house	13.83^**^	2.94	24.73
Medical supply inadequate	Medical supply adequate	46.70^***^	37.30	56.09
Two-year before study leave	One year before study leave	23.25^***^	13.86	32.64
Unsupportive management	Supportive management	21.80^***^	12.82	30.78
Heavy workload	Normal workload	22.10^***^	14.12	30.08

^***^, ^**^, ^*^ ==> *Significant at 1%, 5%, 10% level; CI: Confidence interval*

### Interaction of preference

We have conducted an interaction of preference with sex and age. We used a dummy variable for being male and found no significant interaction between being male and female. Similarly, we have used “young students” aged 21 to 24 years dummy to compare with adult students (25–28 years). We have not found a significant interaction between being young and preference for attributes except for service years. Young medical students prefer one service year to adult students (additional file 1).

## Discussion

Salary was the most important attribute for graduating medical students to work in rural setup. Increasing salary by 25% and 50% from the base (current) salary increased the probability of job take-up by 26.21 and 49.05 percentage points, respectively. Similarly, in choosing health sector job postings, medical students strongly preferred increasing salary levels in the Uganda study [49]. Therefore, posting jobs with increased salaries could potentially increase medical students' rural job take-up rate.

The availability of adequate drugs and medical supplies is the most important attribute for respondents in class 2. In addition, it is the second most important attribute from the pooled dataset following salary. The finding is supported by previous studies, which showed the availability of adequate drugs, equipment, and supplies were the key factors of the job preferred by medical students [45, 47, 49]. Another study in Zambia also described that improvement in facility quality could support health works attraction and retention [62]. The finding was also supported by a study in Vietnam, where medical students value non-financial incentives much more than financial incentives [48]. By forecasting the effectiveness of different policies, the availability of adequate drugs, equipment, and supply in the facility was the most efficient way to increase the take-up rate of the job by 46.7 percentage points to recruit health workers. Similarly, it was the nonwage attribute with the biggest impact on the share of healthcare workers' job uptake in a previous study done in Ethiopia [32]. Improving the availability of adequate drugs and medical supply not only improved patient outcome but also boost medical doctors’ preference to work in rural setting.

Education opportunities after a one-year service had increased students' job preference nearly by a quarter. Students were willing to accept an additional 1998 ETB (48.7 USD) per month instead of an early education opportunity. The finding is supported by a study conducted on medical students in Uganda, which revealed that early education opportunity is preferred [49]. Making job postings with a one-year service education allowance for newly graduated physicians could increase the probability of job uptake by 23.25 percentage points. A DCE study to retain regionalized health personnel in Burkina Faso suggested length of commitment strongly influenced the choice of packages [63]. Therefore, expanding education and professional development opportunities in rural setting could improve the medical professionals preference to work in rural areas.

The availability of supportive management could increase the probability of rural job uptake by 21.80 percentage points in comparison to working in an unfavorable management setup. The finding is also supported by a study conducted in Ghana, where supportive management was most strongly associated with the job preference of medical students [47]. Similarly, the study conducted in Uganda stated, supportive facility managers were important to the student in determining where they would prefer to work [49]. Students were willing to accept around 1868 ETB (45.6 USD) per month to take a facility with unsupportive management instead of supportive.

Regarding workload, medical professionals prefer to work on a normal hour. A facility with a normal workload has a 22.10 percentage points higher job take-up rate than medical students. Contrarily, a study conducted in Tanzania found that decreasing workload does not have a significant effect for unknown reasons [45]. Graduating medical students were willing to accept compensation of 1895 ETB (46.2 USD) per month as compensation of heavy workload than a job with a normal workload.

Even though there is preference heterogeneity between medical students, housing is the least important characteristics for choosing rural jobs. students were willing to accept 1135 ETB (27.7 USD) per month instead for superior housing allowance. The study in Tanzania explained that offers of decent housing appear not important for medical students to prefer job posting and not very highly valued attribute [45]. Provision of superior housing instead of basic increases the probability of job take-up by only 13.8 percentage points, which was the least effect on the probability of job take-up. The study by Peter C Rockers and colleagues in Uganda also found medical students were not express a preference for the provision of actual housing [49]. Therefore, the government should focus more on the most important attributes (improving salary and availability of medical supplies) to increase the uptake rate of rural job listings by medical students.

## Limitations of the study

Even though DCE is a powerful tool with high positive predictive value to predict real-life choices, a hypothetical bias could also affect the estimates. The study used a hypothetical scenario to estimate the population's stated preference, which might not be similar to the actual choice behaviour [64].

## Conclusions

The graduate medical students prefer a rural job with a high salary, superior housing, lesser year of commitment before study leave. In addition, respondents prefer the rural health facility to have supportive management, a normal workload, and fully available drug and medical equipment as per the standard. Moreover, medical students have preferences heterogeneity for the attributes. Therefore, policymakers should consider the medical students preference and preference heterogeneity to incentivize medical professionals to work in rural settings.

## Supplementary Information

**Additional file 1.**

## Data Availability

The datasets used and/or analyzed during the current study are available from the corresponding author on a reasonable request.

## Abbreviations

BDU: Bahir-Dar University
DCE: Discrete Choice Experiment
DTU: Debre Tabor University
FMoH: Federal Ministry of Health
GPs: General Practitioners
HRH: Human Resources for Health
HSTP: Health Sector Transformation Plan
LMICs: Low- and Middle-Income Countries
MIXL: Mixed Logit-model
NGOs: Non-Governmental Organizations
OMEPs: Orthogonal Main Effects Plans
UOG: University of Gondar
WHO: World Health Organization
WTA: Willingness to accept
