# Supplementary material for: Rural job preferences of graduate class medical students in Ethiopia—a discrete choice experiment (DCE)
Source: BMC Med Educ. 2023 Mar 13;23:155. doi: 10.1186/s12909-023-04133-3 (PMC10009985; doi:10.1186/s12909-023-04133-3)
Supplement: Supplementary file 1 — Additional file 1. [file 12909_2023_4133_MOESM1_ESM.docx]

Additional file 1: Interaction of independent variables with preference

Sex interaction: (1=male, 0=female)

Age interaction: (1= Young (21-24 years), 0= Adults (25-28))
